# Episodic Memories in Anxiety Disorders: Clinical Implications

**DOI:** 10.3389/fnbeh.2014.00131

**Published:** 2014-04-24

**Authors:** Armin Zlomuzica, Dorothea Dere, Alla Machulska, Dirk Adolph, Ekrem Dere, Jürgen Margraf

**Affiliations:** ^1^Mental Health Research and Treatment Center, Ruhr-Universität Bochum, Bochum, Germany; ^2^Center for Psychological Consultation and Psychotherapy, Georg-August University Göttingen, Göttingen, Germany; ^3^UMR 7102, Neurobiologie des Processus Adaptatifs, Université Pierre et Marie Curie, Paris, France

**Keywords:** episodic memory, autobiographical memory, cognitive behavioral therapy, anxiety disorders, explicit memory, post-traumatic stress disorder, panic disorder, obsessive–compulsive disorder

## Abstract

The aim of this review is to summarize research on the emerging role of episodic memories in the context of anxiety disorders (AD). The available literature on explicit, autobiographical, and episodic memory function in AD including neuroimaging studies is critically discussed. We describe the methodological diversity of episodic memory research in AD and discuss the need for novel tests to measure episodic memory in a clinical setting. We argue that alterations in episodic memory functions might contribute to the etiology of AD. We further explain why future research on the interplay between episodic memory function and emotional disorders as well as its neuroanatomical foundations offers the promise to increase the effectiveness of modern psychological treatments. We conclude that one major task is to develop methods and training programs that might help patients suffering from AD to better understand, interpret, and possibly actively use their episodic memories in a way that would support therapeutic interventions and counteract the occurrence of symptoms.

## Introduction

### Diagnostic criteria of anxiety disorders

The diagnostic criteria for the classification of anxiety disorders (AD) have been steadily refined during the last few decades. AD are debilitating mental disorders that include specific phobia, generalized anxiety disorder (GAD), social phobia, and panic disorder (PD) (according to DSM-5 criteria) as well as post-traumatic stress disorder (PTSD) and obsessive–compulsive disorder (OCD) (according to the ICD-10 criteria, which include PTSD and OCD in the group of AD). Some common symptoms of AD are excessive rumination, worrying, fear about the future, and active and passive avoidance behavior of the real or imagined fearful stimuli, situations, or events. It is very likely that operant and classical fear conditioning contribute strongly to the development, persistence, and generalization of inappropriate anxiety responses and that AD can be most parsimoniously explained by the principles of learning theory (Bouton et al., 2001).

### Cognitive and learning theories of anxiety disorders

The investigation of cognitive abilities in patients with AD has attracted increasing interest in the last decades. A great deal of clinical research has been directed toward a better understanding of mechanisms underlying changes in explicit and autobiographical memory in highly anxious individuals and patients with AD. These studies have been considerably influenced by cognitive theories of AD (Clark, 1999; Clark and Beck, 2010).

A central element of cognitive theories of AD is the proposition that the negative beliefs and covert cognitive avoidance behavior associated with AD are a result of the patient’s misinterpretation of internal and external stimuli (e.g., the behavior of other individuals, fear associated stimuli from the environment, interoceptive physical sensations, and/or mental events) as being highly dangerous (Bouton et al., 2001; Mathews and MacLeod, 2005). Furthermore, avoidance behavior is maintained because anxiety patients tend to selectively retrieve (personally relevant) information from the past, and in particular those information, which confirm their negative interpretation of current or anticipated situations (Clark, 1999). Likewise, learning theories emphasize the importance of analyzing personally relevant autobiographical memories in anxiety patients (e.g., Öst and Hugdahl, 1981). The examination of the patient’s retrospective memory can reveal the fear conditioning events that have led to the disorder and help to explain the development of negative beliefs and avoidance behavior in these individuals (Öst and Hugdahl, 1981).

## The Relation of Autobiographical and Episodic Memory Retrieval and Anxiety-Related Symptoms

One possible factor that potentially contributes to the maintenance of negative beliefs, maladaptive emotional responses, and avoidance behavior is the dysfunctional retrieval of past experiences from autobiographical and episodic memory (Clark, 1999; Mathews and MacLeod, 2005). It is proposed that AD patients tend to explicitly re-experience past phobic experiences when confronted with the phobic stimulus, which in turn potentiates the fear responses (Fehm and Margraf, 2002; de Quervain and Margraf, 2008). Since exposure to the phobic stimulus is the rationale of exposure-based therapies in AD excessive retrieval of aversive multimodal memories may be even disadvantageous in the context of such an intervention. Therefore, the modulation and interruption of this “vicious circle” is a major focus of novel approaches in the optimization of exposure-based treatments (Fehm and Margraf, 2002; de Quervain and Margraf, 2008).

In contrast to biased retrieval of highly emotional events and stimuli in AD, a growing body of evidence, for instance from neuropsychological investigations, has shown that individuals with clinical levels of anxiety exhibit impaired episodic memory for neutral, emotionally irrelevant information (Airaksinen et al., 2005). These findings suggest that specific characteristics in the storage and retrieval of episodic memory in AD depend on the emotional valence of the processed information. The assessment of everyday’s episodic memory in the course of AD pathology can provide important clues on the patient’s level of cognitive functioning. Normal cognitive functioning is a prerequisite for a number of therapeutic interventions, including cognitive behavioral therapy, and could also be used as an indicator of treatment prognosis and guidance for treatment planning (Tabarés-Seisdedos et al., 2008; Mur et al., 2009).

In sum, a great deal of evidence suggests that highly anxious individuals and patients with AD show systematic changes in explicit and/or episodic memories. However, a closer inspection of the literature reveals several methodological and conceptual issues regarding the definition and measurement of episodic memories, which makes the interpretation of current findings difficult.

## Review Outline

The outline of this review is as follows: first, we discuss the different methods and definitions used to operationalize episodic memory. This enables us to provide a working definition of episodic memory that captures the core elements of the concept and that can be used in clinical studies. Based on this, we will provide a brief critical review of the current state of research on the changes in episodic memories in highly anxious individuals and patients with different AD, i.e., PD, specific phobia, social phobia, PTSD, GAD, and OCD. We will also give an update on studies investigating the neurobiological underpinnings of episodic memory functions in the context of AD. Finally, we will discuss the significance of current episodic memory research for the etiology and treatment of AD.

Our overview is based on evidence accumulated from two major lines of research: (i) studies assessing explicit and autobiographical memories for emotionally loaded and/or disorder congruent information and (ii) investigations on episodic memories for neutral information (as revealed predominantly in the context of neuropsychological investigations) in different AD and highly anxious individuals. Whereas a comprehensive overview of the findings is beyond the scope of this review and can be found elsewhere (McNally, 1997; Coles and Heimberg, 2002; Williams et al., 2007; Mitte, 2008), our central aim is rather to draw conclusions from the findings on the interplay between episodic memory and anxiety. We will delineate that, due to methodological and conceptual issues, current research on episodic memory in clinical settings is still inconclusive. In order to produce meaningful results, clinical research should integrate recent methodological and conceptual refinements from the field of behavioral neuroscience (e.g., Kinugawa et al., 2013; Pause et al., 2013, this research topic; Pause et al., 2010). A more systematic research of episodic memory in AD will be valuable to increase our understanding of the core pathology and mechanisms underlying successful treatment of AD.

## The Concept of Episodic Memory: Past and Present

Before summarizing previous findings on episodic memory functions in anxiety, it is essential to dissect the definition of episodic memory and how the concept of episodic memory, coined by Endel Tulving in the early 1970s (Tulving, 1972), has gradually changed and extended during the last decades (Squire, 2004).

PubMed search using the key words “episodic memory” yielded more than 6200 hits. A careful examination of this literature revealed that there is still no common consensus on the most important core elements of episodic memory and how it should be measured. In fact, numerous working definitions of episodic memory have been proposed. Unfortunately, they are very divergent and sometimes contradictory.

In the early 70s, Tulving (1972) introduced the concept of episodic memory as a system that receives and stores multimodal information about past personal events and their spatial and temporal context. More recent definitions postulate that EM is associated with autonoetic awareness, which describes a feeling that one is remembering something that has happened to oneself, is not happening presently, and is part of one’s personal history (Tulving, 2002; Hampton and Schwartz, 2004; Suddendorf and Corballis, 2007).

Humans are using past experiences (episodic memory) to anticipate future events and plan their actions in these imagined scenarios (prospective memory). Thus, an important extension of the initial definition of episodic memory was that episodic memory is not only concerned with the recollection of past experiences, but also implies one’s capability to imagine or pre-experience events, which may potentially happen in the future (Harris, 1984; Atance and O’Neill, 2001). The latter is referred to as episodic future thinking or prospective memory (Brandimonte et al., 1996).

Most importantly, both subsystems co-exist within the episodic memory system. For example, we remember engaging in a certain action (e.g., meeting a friend in a bar) at a specific place and time (retrospective memory), but we are also likely able to remember to initiate the same action again at a specific time-point within a spatially well-defined location (e.g., meeting another friend in a cafe). Findings from various lesion, pharmacological, and neuroimaging studies implicate that the brain structures mediating retrospective and prospective episodic memory processes are closely linked (Addis et al., 2007). The medial prefrontal cortex and the posteromedial parietal cortex as well as the medial temporal lobes seem to represent the most critical structures for these processes (Addis et al., 2007; Szpunar et al., 2007).

## Explicit Memory Bias in Anxiety Disorders

As outlined above, a great deal of research on amnestic functions in patients with AD was conducted to test the predictions made by cognitive theories of AD (Bower and Cohen, 1982; Beck et al., 1985; Mogg et al., 1987; Bradley et al., 1995). Although these theories are grounded on marginally different models, they all proposed an enhanced implicit and/or explicit retrieval (defined as implicit/explicit memory bias) of disorder-specific information as a crucial factor contributing to the maintenance of anxiety pathology. In other words, patients with AD are more likely to retrieve memories that contain disorder-relevant threatening content as compared to memories bearing disorder-irrelevant neutral or positively valenced contents. In Table 1 studies on explicit memory bias in different AD and their major findings are summarized according to the sample characteristics, diagnostic method, and stimulus materials used.

**Table 1:** **Diagnostic methods and findings from studies assessing episodic memory for emotional and/or disorder-related material in different AD**.

Disorder	Sample characteristics	Diagnostic method(s)	Stimulus material	Memory effect(s)	Reference
**PD and agoraphobia**	PD patients and agoraphobia vs. controls	Free recall	Panic-related vs. neutral passages or threatening vs. neutral words	Enhanced memory for panic-related passages and threatening words in PD	Nunn et al. (1984)
			Anxiety-, panic-, social-, physical-threat words	Enhanced memory for all word types in PD	McNally et al. (1989), Becker et al. (1994), Becker et al. (1999), Cloitre and Liebowitz (1991), Beck et al. (1992), Lundh et al. (1997)
	PD patients vs. controls	Free recall	PD-related vs. pleasant vs. unpleasant words	Enhanced memory for PD-related words in PD	Becker et al. (1994)
		Recognition	Panic-relevant vs. neutral words	Enhanced discrimination and faster reaction times for panic words in PD	Pauli et al. (2005)
	PD patients vs. GAD patients vs. controls	Free recall	Threatening vs. neutral passages or words	No between-group effects	Pickles and van den Broek (1988)
**PTSD**	Crime victims with acute PTSD vs. controls	Free recall	Trauma-related vs. positive and neutral words	Enhanced memory for trauma-related words in PTSD	Paunovi et al. (2002)
	Holocaust survivors with vs. without PTSD vs. controls	Paired associate recall	Holocaust-related vs. neutral words	(a) Enhanced recall of Holocaust-related words in PTSD	Golier et al. (2003)
				(b) Poorer paired associate recall in PTSD	
	War veterans with vs. without PTSD	Free recall; recognition	Combat words (specific or general), other trauma-related vs. neutral words	Enhanced memory for emotional words in PTSD	Vrana et al. (1995)
		Cued recall	Combat, social-threat, vs. positive and negative words	Impaired memory for all word categories in PTSD except for combat words	Zeitlin and McNally (1991)
**Specific phobia**	Spider phobics vs. controls	Recall	Spider-related vs. neutral words	(a) Enhanced memory in spider phobics	Rusted and Dighton (1991), Watts and Coyle (1992)
				(b) Impaired memory in spider phobics	Watts and Dalgleish (1991)
	Spider phobics vs. other specific phobia vs. controls	Free recall; recognition	Video clips of spiders	No group difference on recall or recognition	Thorpe and Salkovskis (2000)
**Social Phobia (SP)**	High social anxiety vs. low social anxiety	Face recognition	Happy vs. angry faces	(a) No effect on recognition in high social anxiety	D’Argembeau et al. (2003)
				(b) Enhanced recognition for happy faces in low social anxiety	
		Recall	Positive social vs. negative social vs. neutral passages	No group effect of stimulus material on recall	Brendle and Wenzel (2004)
			Positive, negative vs. neutral video vignettes		Wenzel et al. (2005)
		Cued recall	Positive vs. neutral vs. social-threat vs. physical-threat words		Lundh and Öst (1997)
	Social phobics vs. controls	Face recognition	Critical vs. accepting faces	(a) Overall enhanced memory for faces in social phobia	Lundh and Öst (1996), Foa et al. (2000), Coles and Heimberg (2005)
				(b) Enhanced memory for critical faces in social phobia	
				(c) Overall impaired memory for faces in social phobia	Pérez-López and Woody (2001)
	Social phobia patients, high social anxiety vs. controls	Free recall	Social phobic vs. neutral verbal stimuli	Enhanced recall for affectively valenced words (positive and threat) in all groups	Cloitre et al. (1995), Lundh and Öst (1997), Rapee et al. (1994), Rinck and Becker (2005)
**GAD**	GAD vs. SP vs. controls	Free recall	GAD-relevant vs. social anxiety-relevant vs. pleasant vs. neutral words	(a) No group effect	Becker et al. (1999)
				(b) Better recall for positive words in all groups	
	GAD vs. depressed vs. controls		Anxiety-relevant vs. depression-relevant vs. positive vs. neutral words	(a) Enhanced recall of depression-relevant words in depressed patients	Bradley et al. (1995)
				(b) No effect of stimulus material for GAD patients	
	GAD vs. controls	Recognition	Threat-relevant vs. neutral words	(a) No group effect	MacLeod and McLaughlin (1995)
				(b) More recognition of threat-words in both groups	
		Free recall	Ideographically selected threat words	Enhanced recall of thread words in GAD	Coles et al. (2007)
**OCD**	OCD patients (with contamination fears) vs. anxious and healthy controls	Free recall	Contaminated vs. clean objects	(a) Enhanced recall of contaminated objects in OCD	Radomsky and Rachman (1999)
				(b) No group effect on recall of which tissue has touched the objects	
	OCD (wash) vs. OCD (checking) vs. socially anxious patients vs. healthy controls			(a) No group effect for recall of contaminated objects	Ceschi et al. (2003)
				(b) Enhanced recall of which tissue has touched the objects	
	OCD patients with checking behavior vs. controls		Threat-relevant vs. neutral actions	Enhanced recall of previously experienced threat-relevant actions in OCD	Constans et al. (1995)
	OCD patients vs. controls	Directed forgetting paradigm; free recall; recognition	Negative vs. positive vs. neutral words	Impaired ability to forget negative words in OCD	Wilhelm et al. (1996)
	OCD patients vs. anxious and non-anxious controls	Free recall; confidence ratings	Safe vs. unsafe. vs neutral objects	(a) No group effect for memory accuracy	Tolin et al. (2001)
				(b) Decreased confidence for unsafe objects over trials in OCD	
	OCD patients vs. subclinical checkers vs. controls	Initial-cued recall; confidence ratings; recognition	Threat-relevant vs. neutral word pairs	(a) Impaired recognition and recall in OCD patients	Tuna et al. (2005)
				(b) Less confidence about future memory performance in OCD	
				(c) No group effect for threat-relevant stimuli	

## Explicit Memory Bias in Panic Disorder and Agoraphobia

The first studies that aimed to test the hypothesis of an explicit memory bias were performed with patients diagnosed with PD and agoraphobia (see Bower and Cohen, 1982; Nunn et al., 1984). According to DSM-5, PD is characterized by unexpected attacks of fear and anxiety, which in some cases (although not necessarily, see Margraf and Ehlers, 1989) are accompanied by strong activation of the sympathetic nervous system. Panic attacks often become chronic and can occur without clearly identifiable causes (triggers) or on occasions where escape and/or help from other persons would be difficult to obtain (the latter being diagnosed as PD with agoraphobia). PD with and without agoraphobia are believed to be based on biological and psychological processes as well as on psychophysiological interactions (Margraf et al., 1986a; Margraf and Ehlers, 1989; McNally, 1990).

In an early investigation by Nunn et al. (1984), patients with PD and agoraphobia and normal controls were instructed to recall text passages containing both, neutral as well as panic-related material. In a subsequent test performed 5 min later, agoraphobic patients recalled more propositions from the panic-related passages as compared to normal controls. This finding was replicated in a subsequent test where agoraphobic patients and controls had to recall threatening and neutral words (Nunn et al., 1984). Again, a superior retrieval of threat-related words relative to neutral words was found in agoraphobic patients. These findings are in line with cognitive and learning theory models of AD, which assume that agoraphobic patients show increased attention to interoceptive physical perceptions while the patient’s interpretation and evaluation of these perceptions is (at least partially) guided by the retrieval of episodic memories of panic-associated, threatening events (Margraf et al., 1986a,b; Margraf and Ehlers, 1989; Zucker et al., 1989; McNally, 1997; Clark, 1999).

Later on, several studies investigated whether patients with PD and agoraphobia would show an explicit memory bias for threatening and panic-related material (see Table 1). These studies typically employed experimental tasks where anxiety patients and healthy participants were instructed to memorize verbal items that were either neutral or contained threatening and/or panic-relevant information. It was found that PD patients exhibit superior recall and recognition of anxiety words (McNally et al., 1989), panic-associated words (Cloitre and Liebowitz, 1991; Becker et al., 1994, 1999; Pauli et al., 2005), socially relevant words (Beck et al., 1992), and physical-threat-related words (Lundh et al., 1997) when compared with healthy, non anxious controls. However, there are also negative findings in the literature (e.g., Pickles and van den Broek, 1988) as well as studies that suggest that the explicit memory bias in PD is highly dependent on the verbal material used (Becker et al., 1994). Becker et al. (1994) investigated the explicit memory bias in PD patients by using learning material that consisted of pleasant, unpleasant, and PD-related words. Here, patients with PD and normal controls were instructed to memorize these three categories of words and to image a specific, personal scene for each word. After the patients and controls had performed a tactile distractor task, explicit memory for the study items was probed with a free-recall test. While the number of correctly remembered positive and negative words was comparable between groups, PD patients recalled more PD-related words than controls.

## Explicit Memory Bias in Post-Traumatic Stress Disorder, Specific Phobia, and Social Phobia

Similar to the results obtained in PD, findings on explicit memory bias in PTSD have been relatively consistent (see Table 1). For example, by using both free and cued recall tests war veterans who had developed PTSD showed an explicit memory bias for combat words (Zeitlin and McNally, 1991; Vrana et al., 1995). Likewise, PTSD patients, who have experienced quite different types of traumatic events, including crime victims (Paunovi et al., 2002) and Holocaust survivors (Golier et al., 2003), have shown an enhanced processing and retrieval of trauma-related words. In contrast to these findings, however, a number of studies consistently reported poorer explicit memory for disorder-irrelevant information in PTSD patients (reviewed in Isaac et al., 2006; Brewin et al., 2007). These findings suggest that explicit memory alterations in PTSD are not global, affecting both trauma-related and -unrelated material. Moreover, the changes are also not unidirectional (only improvement or impairment of memory), but rather highly selective for the type of learning material (but see also section on “Episodic Memory for Neutral Information in Anxious Individuals and Patients with Anxiety Disorders”).

With respect to explicit memory bias in other phobias, studies were predominantly performed with patients suffering from social or spider phobia. For spider phobia, the results are rather mixed as some studies found an enhanced retrieval of spider-related material (Rusted and Dighton, 1991; Watts and Coyle, 1992) while other studies showed opposite findings (Watts and Dalgleish, 1991) or reported similar performance between spider phobic and non-phobic individuals (Thorpe and Salkovskis, 2000).

The majority of studies found no evidence for an explicit memory bias for social-threat stimuli in people with social phobia or a high level of social anxiety (e.g., Rapee et al., 1994; Cloitre et al., 1995; Lundh and Öst, 1997; Brendle and Wenzel, 2004; Rinck and Becker, 2005; Wenzel et al., 2005; see Table 1). Biased retrieval in social phobia was investigated predominantly by using verbal stimuli (Coles and Heimberg, 2002), which might represent a major reason for the low number of positive results. In fact, some researchers noted that it might be more beneficial to use facial expressions of emotions instead of words to study cognitive biases in social phobia, because facial expressions of emotions in particular are the social cues by which social phobia patients are “captured” (Foa et al., 2000; Chen et al., 2002; Pishyar et al., 2004). Foa et al. (2000) provided evidence that participants with generalized social anxiety indeed show a better overall memory for facial expressions compared to non-anxious controls, but also exhibit an enhanced recognition of negative faces compared to positive faces (see also Lundh and Öst, 1996; Coles and Heimberg, 2005; for similar results). However, it should be noted that several subsequent studies failed to replicate the findings of Foa et al. (2000) and demonstrated no differences in the recognition and recall of threatening faces between socially anxious and non-anxious persons (e.g. D’Argembeau et al., 2003; reviewed in Staugaard, 2010). Furthermore, a poorer memory for facial expressions in socially anxious participants compared to healthy controls was also demonstrated (Pérez-López and Woody, 2001). Hence, irrespective of the methodological approach, a conclusion on possible memory biases for threatening information in social phobia is still not possible and continues to be a matter of extensive research (see Staugaard, 2010; LeMoult and Joormann, 2012).

## Explicit Memory Bias in Generalized Anxiety Disorder and Obsessive–Compulsive Disorder

Given the complexity of GAD, it is not surprising that most studies failed to find an explicit memory bias toward threat-related material in GAD patients (e.g., Bradley et al., 1995; MacLeod and McLaughlin, 1995; Becker et al., 1999; reviewed in Coles and Heimberg, 2002). For instance, among other methodological factors, negative findings might be due to the difficulty of finding specific, disorder-relevant trigger stimuli, associated with the GAD patient’s individual domain of worries (see Coles et al., 2007). Coles et al. (2007) used verbal material with individually selected words of personal relevance to the patient’s worry domain and showed that GAD patients tend to exhibit an explicit memory bias, i.e., a superior recall of threatening words as compared to neutral words.

Compared to other AD, little research exists on explicit memory bias in OCD. The majority of the studies so far, found a bias in explicit memory for disorder congruent word-material in OCD patients (see Table 1) although conflicting results had also been reported (e.g., Tuna et al., 2005). Radomsky and Rachman (1999) reported a better memory for contaminated than for clean stimuli in OCD patients, but not in healthy and anxious controls with comparable memory abilities. In line with these results, Constans et al. (1995) reported that OCD patients with checking rituals showed an enhanced recall of previously experienced threat-related actions as compared to non-anxious participants. In another study, neutral, positive, and negative words were presented to OCD patients with different compulsions and obsessions (Wilhelm et al., 1996). In the context of a directed forgetting paradigm, OCD patients recalled more of the negative words that they were told to forget than neutral words assigned with the forget instruction (Wilhelm et al., 1996). Tolin et al. (2001) used a somewhat different approach and presented objects classified as safe, unsafe, or neutral by the participating OCD patients. In a subsequent memory test, the OCD patients, anxious and non-anxious controls were asked to recall as many of these objects as possible and to state the level of confidence in their memories. Although no differences in the recalled number of objects between the three groups were found, the confidence for the same unsafe objects decreased across trials in the group of OCD patients. In a subsequent study using a larger group of OCD patients and more specific controls, Ceschi et al. (2003) showed that OCD patients with washing and cleaning compulsions did not remember more dirty than clean objects when instructed to recall them freely. However, OCD patients showed a memory bias when asked if an object had been touched with a clean or dirty tissue, as they more often recalled the dirty tissue touching the object. It should be noted that the interpretation of these findings is relatively difficult since OCD is also associated with relatively profound deficits in executive functions (Bannon et al., 2006).

## Explicit Memory Bias in Anxiety Disorders: Summary and Interpretation

In a recent meta-analysis of studies investigating implicit and explicit memory bias in clinically anxious patients and non-clinical samples, effect sizes across all studies point toward a better explicit memory recall of threatening material in patients with AD relative to non-anxious and/or low-anxious controls (Mitte, 2008). Nevertheless, as reviewed above, the evaluation of explicit memory biases in AD is a complex research question and several methodological differences need to be taken into account when interpreting the results. The results are mixed and/or there is little evidence available to draw a definitive conclusion. Furthermore, several factors seem to modulate or even confound the observed effects. The magnitude of the explicit memory bias in anxiety populations is highly dependent on the encoding and retrieval procedure, but also on the time interval interposed between the encoding and recall phase (see Coles and Heimberg, 2002; Mitte, 2008). Most importantly, however, most of the studies conducted so far used words or other verbal stimuli like sentences or stories to study the explicit memory bias in AD (Mitte, 2008). In this regard, it was questioned whether verbal learning leads to cognitive biases to the same extent in different AD (e.g., Wenzel and Holt, 2002; Pishyar et al., 2004). While text passages might induce a more elaborative processing than single words in some AD (Wenzel and Holt, 2002), it certainly would be worth investigating whether explicit memory bias could be reliably replicated when using real-life objects and pictorial material (e.g., information from real-life situations and/or social interactions) as learning stimuli (but see Foa et al., 2000). Although the current discrepancies between studies might be due to the specific nature and the severity of the particular AD, it is also likely that the results are highly dependent on the kind of tasks used to measure explicit memory. This question is of special importance when we consider findings of explicit memory bias in AD as proof of alterations in episodic memory (but see Section “The Need for Novel Episodic Memory Tests in the Clinics”).

## Overgeneralized Autobiographical Memories and Biased Retrieval of Autobiographical Content in Anxiety Disorders

Autobiographical memory is another important memory domain that is conceptually related to episodic memory and has been investigated in emotional disorders. The increasing interest of clinical psychologists in autobiographical memory evolved primarily from reports of overgeneralized autobiographical memory (OGM) in patients with major depression. OGM refers to problems in recalling and reporting on temporally and spatially specific events of one’s own personal past.

Up to now, autobiographical memory has been mainly assessed with the Autobiographical Memory Test (AMT), introduced by Williams and Broadbent (1986). In the AMT, participants are instructed to describe specific memories in response to cue words with different valences. As part of the instructions, participants are told that the remembered episode should be an outstanding event, which happened on a particular day and took place at a certain location. Reported memories are coded with regard to their specificity by an independent rater. Only when the participants provide information about the time and the particular place of the reported event, it is rated as a specific memory. In contrast, if participants report more generic personal memories based on repeated experiences or entire periods in their lives, these memories are classified as OGM. However, it is important to note that most versions of the AMT involve response-time limits of 30 or 60 s and that omissions are in some cases added to the OGM score (Van Vreeswijk and de Wilde, 2004; Griffith et al., 2012). Therefore, one has to consider that any physiological process or pathology involving slowing of reaction times or response latencies can confound the measurement in this task. Aged individuals or persons suffering from a depressive episode as well as patients using anxiolytic drugs are expected to have longer reaction times and response latencies.

Williams and Broadbent (1986) were the first to delineate the relation between psychopathology and OGM. In their seminal study, suicidal patients retrieved autobiographical events in a more general mode as compared to clinical and healthy controls. Later on, Williams et al. (1996) extended their initial findings and demonstrated that compared to controls, suicidal depressed patients also anticipate future events with a similar lack of episodic specificity.

## Overgeneralized Memories in Major Depression and Post-Traumatic Stress Disorder

Overgeneralized autobiographical memory was proved to be a relatively stable characteristic of patients with a major depression. A higher level of OGM has been proposed to be a risk factor that increases the chance to develop major depression (Rawal and Rice, 2012), and is predictive of an unfavorable prognosis (see meta-analysis by Sumner et al., 2010).

The OGM effect is not restricted to individuals with major depression. There is also substantial evidence for OGM in patients with a PTSD (McNally et al., 1994, 1995; Moore and Zoellner, 2007; Sutherland and Bryant, 2007, 2008a; Moradi et al., 2012), cancer survivors (Kangas et al., 2005), and injured individuals with acute stress disorder (Harvey et al., 1998; see Table 2 for summary). Brown et al. (2013) recently demonstrated that PTSD patients show similar deficits in memory specificity when generating future events. However, it should be noted that to date there is no evidence for a causal relationship between OGM and the etiology of major depression and/or PTSD. Nevertheless, it has been proposed that by being overgeneral, patients with emotional disorders might try to avoid the conscious processing of highly emotional material associated with negative personal experiences (Williams et al., 2007). In particular, since the remembrance of specific aversive experiences often evokes mental images, physical symptoms, and negative emotions (Hermans et al., 2006), the retrieval of less specific/detailed memories may be a coping strategy that protects against negative affects (Hermans et al., 2006; Williams et al., 2007). This type of aversive-memory manipulation qualifies as a cognitive avoidance behavior that is associated with a negative long-term outcome: patients continue to maintain impaired affect regulation and show an increased vulnerability for future depressive and anxiety symptoms (Hermans et al., 2006; Kleim and Ehlers, 2008; Watkins, 2008).

**Table 2:** **Findings on OGM and biased autobiographical memory in AD as assessed by different versions of the autobiographical memory test**.

Disorder	Sample characteristics	Material	Memory effect(s)	Reference
**PTSD**	Vietnam combat veterans with PTSD vs. with other disorders vs. without other disorders	Combat-relevant vs. neutral videotape and positive, neutral, and negative cue words	OGM especially after combat-relevant videotapes in PTSD	McNally et al. (1994)
	Veterans with vs. without PTSD vs. controls	Trauma-related vs. positive cue words	(a) OGM in PTSD: fewer specific memories in PTSD than in veterans without PTSD and controls	Moradi et al. (2012)
			(b) Fewer specific memories in veterans without PTSD than in controls	
	Injured individuals with vs. without acute stress disorder (ASD)		(a) OGM for positive cues in ASD	Harvey et al. (1998)
			(b) Correlation with PTSD symptom severity at a 6 months follow-up	
	Patients with vs. without ASD after cancer diagnosis		OGM in patients with ASD	Kangas et al. (2005)
	PTSD vs. trauma-exposed non-PTSD individuals	Positive vs. negative cue words	OGM in PTSD regardless of cue valence	Sutherland and Bryant (2008a)
			More trauma-related memories in response to positive cues	Sutherland and Bryant (2008b)
	PTSD patients		Symptom improvement after cognitive behavioral therapy associated with improved retrieval of specific memories in response to positive cues	Sutherland and Bryant (2007)
	Combat veterans with vs. without PTSD	Neutral cue words	(a) OGM in PTSD	Brown et al. (2013)
			(b) Overgeneral future imaging in PTSD	
**Various anxiety disorders**	AD with a history of major depression vs. AD and major depression in remission vs. AD and current major depression vs. major depression vs. controls	Positive vs. negative cue words	(a) No OGM in AD patients (b) OGM only in patients with a major depression	Wessel et al. (2001)
**GAD**	GAD patients vs. controls	Neutral cue words	(a) More and faster recall of anxiety-evoking memories in GAD patients	Burke and Mathews (1992)
**Social Phobia (SP)**	SP patients with current comorbid depression vs. depression patients without AD vs. controls	Positive vs. negative cue words	No between-group effect on OGM	Heidenreich et al. (2007)
	SP patients vs. controls	Social-threat vs. neutral words	No memory bias toward threat in SP patients	Wenzel et al. (2002)
		Social-threat vs. positive vs. neutral cue words	More retrieval of specific negative memories when cued by social threat in controls	Wenzel et al. (2004)
	High social anxiety vs. low social anxiety	Negative vs. positive cue words	(a) More negative memories in high social anxiety	Krans et al. (2013)
			(b) More social anxiety-related memories and more positive memories in response to negative cues in low social anxiety	
**SP and panic disorder (PD)**	SP patients vs. PD patients vs. controls	Social phobia-related vs. panic-related vs. control cues	(a) Faster recall of autobiographical memories after social phobia-related cues in SP	Wenzel and Cochran (2006)
			(b) Faster recall of autobiographical memories after panic-related cues in PD	
**Specific Phobia**	Spider fearful vs. blood/injury-fearful individuals vs. controls	Spider-related vs. blood/injury-related vs. neutral cue words	Increased retrieval of generally negative memories in anxious individuals	Wenzel et al. (2003)
**OCD**	OCD patients vs. controls	Positive vs. negative cue words	OGM in patients only in the presence of a comorbid MD diagnosis	Wilhelm et al. (1997)

However, it should be noted that apart from PTSD, studies conducted in patients with other AD could not demonstrate an OGM (Burke and Mathews, 1992; Wenzel et al., 2002, 2004; Wenzel and Cochran, 2006; Heidenreich et al., 2007). The presence of a full-blown major depression, as a comorbid factor, or at least some depressive-like symptoms, rather than the AD itself, might be the critical factor that possibly determines whether or not OGM are evident in a given disorder (Wilhelm et al., 1997; Wessel et al., 2001; Williams et al., 2007).

## Biased Autobiographical Memory in Anxiety Disorders

In sum, the data reviewed so far suggest that AD are not directly associated with OGM. To the contrary, there is some evidence indicating that some AD (i.e., PD, specific and social phobia) are characterized by a biased recall of autobiographical memories (see Table 2). In particular, it has been shown that PD patients exhibit a threat-relevant memory bias for autobiographical events. This bias is indicated by a more rapid retrieval and a more accurate reproduction of the content of threat-relevant material after being cued with PD-related thoughts (Wenzel and Cochran, 2006). Likewise, there is some evidence that socially anxious individuals show an enhanced memory of threatening and highly emotional autobiographical material (Wenzel and Cochran, 2006; Krans et al., 2013; reviewed in Morgan, 2010), which is being discussed as a core feature of the psychopathology in social anxiety (Morgan, 2010). Finally, it has been shown that spider- and blood/injury-fearful individuals exhibit an enhanced retrieval of negative autobiographical memories and show a greater specificity in the recall of episodes containing direct encounters with the feared stimuli (Wenzel et al., 2003).

## Autobiographical Memory Bias and Overgeneralized Memories in Anxiety Disorders: Summary and Discussion

Overall, the literature on autobiographical memory function in AD is still inconclusive and certainly more research on this issue is needed. Some evidence is available for biases in autobiographical memory, but the results are mixed and are exclusively derived from patients with PD and specific or social phobia. Prior to a final evaluation of these studies, it is necessary to briefly discuss the pitfalls and drawbacks of the methods used in these studies. Most of the findings so far have been obtained with the AMT. While the AMT is a gold standard in research, in a recent review by Griffith et al. (2012), the authors list numerous critical issues, all of which can contribute to differences in the calculation and interpretation of AMT scores. According to this review it can be concluded (see Griffith et al., 2012 for more details) that several pitfalls listed below might have led to misleading results in the existing research literature: (I) the execution of the AMT is not standardized and differs across various studies. For example, cue words are presented orally, visually, both orally and visually, or in written form, sometimes by the researcher, sometimes within a computerized version. (II) The word lists used in the AMT were not standardized, but varied across studies in the number of items and word-type, degree of imageability, emotionality, and personal relevance of the retrieval cues. (III) Whether or not response-time limits were applied varied across studies. However, this factor has a profound impact on the way memories are encoded or retrieved. (IV) The interpretation of the AMT results was critically dependent on the study-specific scoring procedure as well as on the handling and scoring of omissions (see also Van Vreeswijk and de Wilde, 2004). Moscovitch et al. (2011) criticized the use of the AMT in the context of social phobia, since the standardized single word cues in the AMT are not sufficient to activate vivid mental images of autobiographical memories in socially anxious participants. Furthermore, the coding system of the AMT might oversimplify the complexity of remembered memories and might subsequently force raters to arbitrarily categorize a reported memory as either specific or general. Moscovitch et al. (2011) proposed that a dimensional approach would be more reasonable since the binary view on specific memories as existent or absent excludes the possibility of measuring partially accessible specific autobiographical memories.

Another important issue that should also be stressed is that a subject’s performance in the AMT does not allow conclusions about whether the person accurately recollects his/her previous experiences. In its current form, the AMT does not involve a procedure that probes memory accuracy (Brewin et al., 2010). Moreover, autobiographical experiences are likely to be retrieved and narrated repeatedly, and due to their constructive and dynamic nature, there might be changes in the contents as well as the stability of a memory across time (e.g., due to reconsolidation and interference phenomena, see Schacter et al., 1998; Walker et al., 2003; Earles et al., 2008). Thus, several methodological difficulties are associated with ATM based research, and one should be cautious in drawing the definitive conclusion that autobiographical memory is not affected in a given AD.

## Episodic Memory for Neutral Information in Anxious Individuals and Patients with Anxiety Disorders

While the studies on explicit and autobiographical memory bias stress change in the recall of disorder congruent information in AD, a common secondary finding of these studies is that the recall of neutral or emotionally irrelevant material seems to be largely unaffected in AD. Can we thus conclude that global memory performance, and in particular episodic memory for neutral information, is not affected in clinically anxious people?

Studies on neurocognitive profiles in anxiety patients only partially support this assumption as outlined in the following sections.

## Episodic Memory for Neutral Information in Anxiety Disorders

An overview of studies, which assessed episodic memory for neutral information in AD is given in Table 3. While some studies reported deficits in the recall of neutral verbal information in both social phobia and PD (Lucas et al., 1991; Asmundson et al., 1994; Airaksinen et al., 2005), other studies found no evidence for such impairments in social phobia (Sachs et al., 2004; Sutterby and Bedwell, 2012) and/or PD (Gladsjo et al., 1998; Purcell et al., 1998; Boldrini et al., 2005; see Table 3).

**Table 3:** **Diagnostic methods and findings from studies assessing episodic memory for neutral and/or disorder-unrelated material in different AD and highly-anxious individuals**.

Anxiety disorder and sample characteristics	Diagnostic method(s) and/or stimulus material	Memory effect(s)	Reference
**Population-based sample comprising individuals with PD with and without agoraphobia, social phobia, GAD, OCD, specific phobia vs. healthy controls**	Free recall of neutral words	(a) Impaired recall in PD with and without agoraphobia, social phobia, and OCD	Airaksinen et al. (2005)
		(b) No effect in GAD and specific phobia	
**PD patients vs. controls**	Wechsler Memory Test (WMT); selective reminding; verbal and visual recall	(a) Impaired memory for visual, but not verbal memory in the WMT	Lucas et al. (1991)
		(b) Impaired recall of verbal and visual items	
	Free and cued recall (California Verbal Learning Test; CVLT); visual recognition (Continuous Visual Memory Test; CVMT); Verbal and face recognition (Warrington Recognition Memory Test; WRMT)	(a) No group differences (b) No effect of symptom severity on memory performance	Gladsjo et al. (1998)
**PD patients vs. social phobia patients vs. controls**	Neuropsychological test battery (verbal learning and memory, visual memory)	(a) Reduced performance in verbal learning and memory in PD and social phobia	Asmundson et al. (1994)
		(b) Inhibited free recall in PD	
		(c) No group effect on visual memory	
**Generalized social phobia vs. controls**	Verbal and visual immediate and delayed recall of word lists and visual scenes	No group effect	Sutterby and Bedwell (2012)
**PTSD patients vs. trauma-exposed individuals vs. healthy controls**	Different paradigms for the assessment of episodic memory for neutral information: immediate and delayed verbal recall; visual recognition memory; auditory–verbal recall (Rey Auditory–Verbal Learning Test; RAVL); visuospatial recall; CVLT; different subtests from Wechsler Memory Scale; Visual memory: Rey–Osterrieth Complex Figures Test; ROCF) etc.	Overall mixed results with respect to episodic memory for neutral (verbal and visual) information	Reviewed in Brewin et al. (2007) and Isaac et al. (2006)
**OCD patients vs. PD patients vs. depressed individuals vs. controls**	Spatial short-term memory (Corsi Blocks); visual memory: recall and recognition	(a) Impaired spatial recognition in OCD	Purcell et al. (1998)
		(b) No impairments in PD or depressed individuals	
**OCD patients vs. PD patients with agoraphobia vs. controls**	Spatial memory (Corsi Block Tapping Task); verbal memory (Digit Span, Buschke–Fuld Selective Reminding Test)	(a) Impaired verbal memory in OCD	Boldrini et al. (2005)
		(b) Impaired spatial memory in OCD and PD	
**OCD patients with checking behavior vs. controls**	Digit span test; immediate and delayed recall and recognition etc.	Impaired recall and recognition in OCD	Tallis et al. (1999), Savage et al. (1999), reviewed in Greisberg and McKay (2003)
**OCD unmedicated patients vs. controls**	ROCF; non-verbal recall	Impaired recall in OCD (depending on symptom severity and regional cerebral blood flow in the right thalamus)	Lacerda et al. (2003)
**OCD patients vs. controls**	Word lists; ROCF; free recall and recognition of verbal and visual stimuli (e.g., faces, figures) etc.	(a) Verbal and visual episodic memory impairments (b) Mediation of memory performance by organizational strategies (c) No effect for face recognition	Savage et al. (2000), Penadés et al. (2005) Reviewed in Olley et al. (2007)
**OCD patients before vs. after treatment with selective serotonin reuptake inhibitors (4 months)**	Non-verbal recall (RCFT)	Improvement of immediate- and delayed-recall after treatment	Kang et al. (2003)
**Medicated vs. unmedicated OCD patients**	Delayed verbal recall (RAVL); procedural memory (computerized procedural memory test)	No group effect	Mataix-Cols et al. (2002)
**GAD patients aged >60 (before and after treatment with escitalopram for 12 weeks) vs. controls aged >60**	Immediate and delayed recall of word lists, stories, and figures	(a) Memory impairment in GAD patients before treatment	Butters et al. (2011)
		(b) Memory improvement in delayed memory after treatment in GAD patients with low cognitive scores in baseline	
		(c) Strong relationship between anxiety and memory improvement	

Likewise, while extensive research on episodic memory functions has been conducted in patients with PTSD, recent reviews and meta-analysis of the existing literature on episodic, non-trauma-related memory functioning in individuals with PTSD conclude that there is no definite answer to the question (Stein et al., 2002; Neylan et al., 2004; Isaac et al., 2006), or that the association between PTSD and memory impairment appears to be only moderate (Brewin et al., 2007). A series of methodological difficulties arise when interpreting episodic memory functioning in PTSD (see Isaac et al., 2006; Brewin et al., 2007). Memory for emotionally neutral verbal information in PTSD was poorer than the recall for visual stimuli (Vasterling et al., 2002; Brewin et al., 2007). Most importantly, the difficulty of co-morbidity in the patient samples, lack of clear differentiation between PTSD patients who vary with regard to time since trauma, and deficits in attention and executive functions in PTSD (which represent core criteria for the diagnosis of PTSD according to DSM-5), could *per se* have contributed to the findings of impaired episodic memory in PTSD (see Isaac et al., 2006).

Several neuropsychological studies exist in OCD patients (see Greisberg and McKay, 2003 for a review of the literature). While profound deficits in episodic memory functions in OCD were reported (Savage et al., 1999; Tallis et al., 1999; Kang et al., 2003; Kuelz et al., 2004; Boldrini et al., 2005; reviewed in Greisberg and McKay, 2003), negative findings had also been obtained (Mataix-Cols et al., 2002; Bohne et al., 2005). As outlined above, a major factor which may account for the (episodic) memory deficits demonstrated in OCD is a concomitant impairment in executive functions due to frontal lobe dysfunction (Lacerda et al., 2003; Van den Heuvel et al., 2005; Olley et al., 2007). As a consequence, some researchers attempted to dissect the executive component from episodic memory performance in OCD. By using this approach, it was shown that an increased level of organization (in addition to the recall of information) leads to deficits in episodic memory in OCD, whereas the recall of information under controlled and well-organized conditions seems to be unaffected (Savage et al., 2000; Greisberg and McKay, 2003; Penadés et al., 2005). Thus, the memory performance of OCD patients is strongly dependent on task demands. Detrimental effects on episodic memory are more likely to occur in OCD when patients are forced to use organizational strategies during learning (Savage et al., 2000; Greisberg and McKay, 2003; Penadés et al., 2005).

Empirical evidence on episodic memory functions in GAD is relatively scarce (but see Airaksinen et al., 2005). In one of the rare comprehensive evaluations of neurocognitive functions in GAD, deficits in episodic memory (delayed visual and verbal recall) have been reported only for older adults (mean age 71.6 years) diagnosed with GAD (Butters et al., 2011). Interestingly, a 12-week lasting treatment with escitalopram (Cipralex^®^) improved memory in the delayed verbal memory test in GAD patients with low cognitive scores. In one of the most detailed existing studies, Airaksinen et al. (2005) assessed cognitive functions and specifically episodic memory in population-based samples of persons suffering from an AD. In contrast to other studies, participants were recruited from a population-based sample of persons meeting the DSM-IV criteria for an AD. Overall, their results point toward significant deficits in episodic memory in all subgroups of AD, as measured by both free and cued recall of verbal information. However, episodic memory impairments were more profound in PD and social phobia patients than in any other AD (Airaksinen et al., 2005). The impairment in episodic memory was persistent even when controlling concomitant depressive disorder, alcohol dependence/abuse, or psychopharmacological treatment as covariates. While their outcome somehow suggests that AD indeed have a negative influence on episodic memory functioning (Airaksinen et al., 2005), such a conclusion might be premature, as discussed in the following sections.

## The Neuroanatomy of Episodic Memory in Anxiety Disorders

Surprisingly, little research has been conducted to identify the neuronal mechanism, which underlies changes in episodic memory functions in the context of an AD. Most of the studies in this field have been conducted in patients diagnosed with PTSD and trauma-exposed subjects without a PTSD diagnosis. By using various imaging techniques [functional magnetic resonance imaging (fMRI) and positron emission tomography (PET)], specific neuronal activation patterns have been described in brain regions previously associated with episodic memory encoding and retrieval, i.e., the hippocampus, amygdala, and the prefrontal cortex. For example, Brohawn et al. (2010) assessed recognition memory for trauma-related, neutral and positive pictures in PTSD patients and trauma-exposed non-PTSD subjects by using an fMRI approach. They found increased amygdala activation during the encoding of negative (vs. neutral) pictures as well as exaggerated hippocampal activation during recognition of trauma-related pictures in PTSD diagnosed patients. Interestingly, the degree of amygdala activation was positively correlated with current PTSD symptom severity while the amount of hippocampal activation during retrieval was greater in PTSD diagnosed individuals compared to non-PTSD subjects. Dickie et al. (2008) assessed the memory for neutral and fearful face expressions in PTSD patients. They found a negative correlation between the patient’s symptom severity (as assessed by Clinically Administered PTSD Scale scores) and memory performance as well as ventral medial prefrontal cortex activity elicited by the subsequently forgotten faces. Similar activation patterns during the storage and retrieval of emotionally loaded episodic memories in PTSD patients have been reported in other studies (Bremner et al., 2003a; Thomaes et al., 2009; Whalley et al., 2009; Hayes et al., 2011). There is also evidence that PTSD patients exhibit a diminished hippocampal activation during encoding (Bremner et al., 2003b), as well as an abnormal regional blood flow response (by using PET) in the hippocampus during the recollection (Shin et al., 2004) of neutral material. In a recent, quite interesting, study by Dickie et al. (2011), changes in neuronal activation during the performance in an emotional face memory encoding task have been reported in patients who recovered from PTSD symptoms. In particular, PTSD patients showing symptom improvement over several months exhibited specific changes in memory-related activations in the hippocampal regions and the subgenual anterior cingulate cortex, suggesting that learning induced neuronal activity might serve as an indicator of PTSD recovery.

To our knowledge, there are only two reports published in which fMRI was recorded in other AD patients during episodic memory performance (Maddock et al., 2003; van Tol et al., 2012). Maddock et al. (2003) demonstrated that patients with PD engage in more intense information processing and deeper encoding when confronted with threat-related stimuli. Most importantly, this increased effort in encoding of threat-related stimuli was associated with higher neuronal activation in the posterior cingulate and dorsolateral prefrontal cortices and with better memory performance for threat-related vs. unrelated stimuli. van Tol et al. (2012) assessed encoding and recognition-associated neuronal activation in AD patients (diagnosed with a PD, social anxiety, and/or GAD), patients with a major depression, comorbid major depression, and healthy controls during an emotional word memory paradigm. Participants were asked to memorize positive, negative, and neutral words and complete a word-recognition task after a short retention interval. Findings from this study point toward a decrease in hippocampal activation during encoding in both patients with major depression and patients diagnosed with an AD. However, specific increase in the activation in the inferior frontal gyrus during the subsequent recognition of previously learned positive material was found exclusively in patients with AD. The observed effects were largely unaffected by medication status and regional brain volume.

Although the evidence available so far is still scarce, findings from neuroimaging studies suggest the presence of a specific and symptom-related activation pattern in brain regions, which are critical for the storage and retrieval of emotionally loaded episodic memories (see Maddock, 1999).

## Autobiographical vs. Episodic Memory: Different Sides of the Same Coin?

The episodic memory system is required for the storage of highly detailed knowledge of past experiences including spatial and temporal context as well as the internal state of the individual during the encoding of the experienced event [including a blueprint of emotions, perceptions, and thoughts in this situation (Dere et al., 2010)]. In contrast, the autobiographical memory system stores parts of the content and context information of an episodic memory and modifies and extends this content information with knowledge from the semantic memory system (reconsolidation–extension phenomenon). In other words, the autobiographical memory system modifies and integrates episodic memories into the individual’s biography that take the form of a semantic biographical memory. The important difference between autobiographical and episodic memories is that autobiographical memory can be de-emotionalized, modified, and extended by semantic knowledge in terms of its content (there is evidence that the content of autobiographical memories changes over time with each retrieval–extension–reconsolidation round). Furthermore, it does not necessarily contain a blueprint of the internal state of the individual during encoding and is therefore not associated with a reliving of the experience during recollection as postulated by Tulving (Tulving, 2002; Dere et al., 2010). Thus, autobiographical memories are semantic memories of biographical facts containing some of the information associated with an episodic memory, but are not associated with phenomenological aspects of remembering a past episode (autonoetic awareness), as was originally proposed by Endel Tulving (Tulving, 2002, but see Conway, 2001). Fivush (2011) argues for a developmental distinction of episodic and autobiographical memory. He describes a developmental model where autobiographical memories are built on episodic representations and bind past events together into a personal history. Within this model, autobiographical memories are supposed to act beyond the episodic memory function and represent the central component for guiding current and future behavior (Fivush, 2011). Given these theoretical distinctions between episodic and autobiographical memory, the AMT should not be applied to measure episodic memory function.

## Autobiographical vs. Verbal Episodic-Like Memory: Same or Different?

The AMT also differs from the assessment of episodic memory through the reproduction of learned lists of items (words, pictures, or faces) in a laboratory setting (Gilboa, 2004; McDermott et al., 2009; Fivush, 2011). The verbal memory test is also not a valid measure of episodic memory function, since it lacks the assessment of the recollection of spatial and temporal information and should be considered as measuring only an episodic-like memory. The neuroanatomy of verbal episodic-like and autobiographical memory has been reported to be only partially overlapping. There were substantial differences in the neuronal activation patterns between verbal episodic-like memories and autobiographical memories (Gilboa, 2004; Burianova and Grady, 2007; McDermott et al., 2009). Additionally, differences in the temporal dynamics related to the retrieval of autobiographical and verbal episodic-like memory allowed a further distinction between these two memory systems (Conway, 2001).

## The Need for Novel Episodic Memory Tests in the Clinics

The majority of studies so far used verbal or in some rare cases visual learning to assess episodic memory for emotional and neutral information in AD. It is doubtful whether commonly used tests of episodic memory function are valid measures of episodic memory in a clinical setting (irrespective of whether they rely on the participants’ ability to learn words and/or photographs and remember these at a later point via recall, cued recall, or recognition test). One problem raised is that such paradigms measure only one isolated core component of episodic memory, rather than subcomponents (the memory for what, where, and when) and the binding of these components into a unified integrated memory episode according to the definition of episodic memory as a multimodal memory system (see Pause et al., 2010, 2013; Plancher et al., 2010). Verbal learning tests, for example, do not explicitly measure the memory for spatial and temporal context information associated with the learning event, and often there is no spatial and temporal component implicated in the way the verbal memory is tested (Pause et al., 2013). As a consequence, various spatiotemporal memory paradigms have recently been developed, allowing the induction and measurement of the core components of an episodic memory (event, spatial, and temporal information) under experimentally well-defined conditions (Pause et al., 2010; Bellassen et al., 2012; Plancher et al., 2012; Kinugawa et al., 2013).

Other researchers yet suggest that much of what we remember in everyday life refers to visual information embedded in scenes and past actions, and thus emphasize the importance of using ecologically valid episodic memory tasks in a laboratory setting (Piolino et al., 2009; Plancher et al., 2010, 2012). Plancher et al. (2012) used a virtual–reality-based environment in a highly ecological fashion to characterize impairments in episodic memory in amnestic mild cognitive impairment (MCI) and Alzheimer’s disease. By using a multimodal approach, the memory for central and perceptual details, spatiotemporal contextual elements, and binding of these elements was assessed in an environment corresponding to real-life experiences (i.e., driving a car through a “virtual” city where different scenes and actions were presented at different locations and at different time-points). The authors found specific episodic memory profiles in amnestic MCI and Alzheimer’s disease. Interestingly the patients’ daily memory complaints were more highly correlated with the performance in the virtual test than with their performance in the classical neuropsychological memory test.

Irish et al. (2011) investigated episodic memory capacity in amnestic patients with MCI by using a battery of experimental tasks with a high relevance to real-world situations. MCI patients displayed impairments across all domains of episodic memory and particularly in the experimental tasks associated with actions significant in everyday functioning, e.g., remembering faces and names, finding a route, and/or remembering typical daily routine sequences (e.g., getting dressed, traveling to work, the weather that day, see Irish et al., 2011). Thus, it can be argued that episodic memory tests in the laboratory setting can capture core elements of everyday episodic memory and that impairments in specifically designed paradigms in the laboratory can be reliable indicators of changes in everyday episodic memory functions in real-life situations.

As discussed in this section, definite conclusions on possible changes in episodic memory for emotional and neutral information in highly anxious individuals and patients with AD cannot be drawn from the existing literature. We argue that there is still a shortage of studies using valid standardized tests to induce episodic (like) memories and their retrieval (but see Pause et al., 2013) under laboratory conditions. Furthermore, the acceptance of verbal and visual memory tasks as a measure of episodic memory functions largely neglects the complexity of the everyday episodic memory since such tests lack ecological validity and thus cannot provide insights into the individual’s level of functioning in real-life situations. There is certainly also the need to develop more reality-based tests that allow a naturalistic assessment of episodic memory in AD that is also useful to monitor changes in response to therapeutic interventions. In this regard, already available virtual reality tests of episodic memory function that have been developed for patients with MCI an Alzheimer’s disease could be adapted to AD patients using disease-related and non-related stimuli (Plancher et al., 2012). Of course the test has to be designed in a way that allows repeated administration in order to evaluate the outcome of pharmacological or therapeutic interventions of episodic memory performance or whether fluctuations in symptom strengths are associated with changes in episodic memory performance.

## The Significance of the Episodic Memory Concept in the Context of AD

Episodic memory is presumed to have an important evolutionary function, which has remained common across different species (Allen and Fortin, 2013). This unique functional feature of the episodic memory system allows organisms to retrieve spatially and temporally specific information about single, unique experiences (Squire, 2004; Allen and Fortin, 2013). Several lines of evidence show that the retrieval of such spatiotemporal specific experiences is highly relevant for both humans as well as non-human animals (Bluck and Alea, 2002; Dere et al., 2006, 2008).

## Episodic Memories, Problem Solving, and Self-Efficacy

Episodic memory is closely related to the level of functioning in daily life (Irish et al., 2011). In order to initiate adaptive behavior in the present or immediate future, humans, as well as animals, are able to retrieve information encoded during specific events and use these informations to form “memory-based” predictions (Allen and Fortin, 2013). In particular, when faced with current and anticipated problems, one’s ability to retrieve specific details from previous personal events can provide important clues for efficient problem solving (Bluck and Alea, 2002; Williams, 2006; Beaman et al., 2007). Empirical evidence supports the link between episodic memory and problem-solving capacity, i.e., episodic simulation and/or higher specificity in autobiographic memory retrieval is associated with higher effectiveness in problem solving (Brown et al., 2012; Vandermorris et al., 2013). Accordingly, deficits in the specific retrieval of past experiences are highly correlated with poor problem solving (Evans et al., 1992; Sutherland and Bryant, 2008a; MacCallum and Bryant, 2010), which has a clinical impact and is therefore displayed in depressed (Goddard et al., 1996), suicidal (Evans et al., 1992; Pollock and Williams, 2001), and traumatized patients (Sutherland and Bryant, 2008b). However, the underlying cognitive mechanisms of the association between deficits in episodic memory functions and pathological conditions are still a matter of research (Williams, 2006; Williams et al., 2007).

There is also substantial evidence that episodic memory functions have therapeutic relevance and might be directly related to psychological treatment outcome. For instance, one of the core interventions in modern psychological therapies (i.e., cognitive behavioral therapies) for the treatment of mental diseases is to train patients to generate specific skills in order to manage current and future problems. Patients are encouraged to learn to reduce ineffective ways of reacting (e.g., showing avoidance or passivity) and instead react to problems effectively by applying new problem-solving skills (Nezu et al., 2003). The latter can be modified within the therapy context via cognitive restructuring procedures, e.g., realistic goal setting, success visualization, and use of previous negative experiences as a clue to solve present and future anticipated problems (Nezu et al., 2003). The capability to retrieve details from one’s own past in a specific way as well as the ability to project oneself into the future and simulate novel events therefore has a potential influence on treatment outcome. Accordingly, instructions aimed at promoting the retrieval of specific positive memories during psychotherapy (i.e., memory specificity training) have recently been associated with greater symptom reduction in older depressed patients (Serrano et al., 2004), adolescents with depression (Neshat-Doost et al., 2013), and inpatients with depressive symptomatology (Raes et al., 2009). Likewise, psychological treatments lead to both symptom improvement and an increased retrieval of specific autobiographical memories in former depressive patients (Williams et al., 2000), patients with complicated grief (MacCallum and Bryant, 2010) as well as PTSD patients (Sutherland and Bryant, 2007). Moreover, Ehlers and Clark (2000) proposed an etiological model of PTSD in which the predictive valence of autobiographical memory capacity for the subsequent development of a PTSD plays a central role (Kleim and Ehlers, 2008). According to this model, successful integration of intrusive traumatic memories into the autobiographical memory base is considered as a critical element for a successful treatment of PTSD (Ehlers and Clark, 2000).

In a very recent study, Brown et al. (2012) suggested that therapy-induced increase in self-efficacy may have a beneficial impact on future directed episodic memory specificity and consequently problem-solving strategies. In their experimental work, they examined whether manipulating self-efficacy in healthy students has an impact on the capacity to recall the past and imagine one self in the future (mental time travel), and whether such manipulation can further influence social problem solving. They showed that compared to individuals in the low self-efficacy group, individuals in the high self-efficacy group generated past and future events with greater specificity and also performed better on social problem-solving indices. It should be noted that the majority of studies on clinical significance of episodic memory functions deal with affective disorders. Apart from PTSD (Sutherland and Bryant, 2007, 2008a), evidence for an association between therapy-induced symptom reduction, problem-solving capacity and episodic memory specificity in AD requires empirical confirmation.

## Excessive Retrieval of Aversive Experiences in the Context of Exposure-Based Treatments

Different lines of evidence suggest that the excessive episodic retrieval of aversive experiences may be disadvantageous and may hamper efficacy of psychotherapeutic interventions. Accordingly, the modulation of excessive retrieval (i.e., reducing excessive retrieval) of aversive episodic memories in anxiety patients might represent a mechanism to enhance the effectiveness of exposure-based treatments. Exposure-based treatments arguably represent the most effective form of a psychological intervention in the treatment of AD (Margraf, 2000). However, inter-individual differences in the treatment benefit during exposure-based therapy exist among anxiety patients. For example, a substantial proportion of anxiety patients shows only partial reduction of anxiety symptoms after exposure therapy and/or displays a high incidence of fear relapse phenomena after the completion of the treatment (Heimberg, 2002; Blanco et al., 2003; Davidson et al., 2004; Cottraux, 2005). It is proposed that exposure to a phobic stimulus not only leads to the activation of stimulus-associated fear memory, but is also connected with the retrieval of explicit fearful memories of past phobic experiences, which in turn enhances the fear responses (Lang, 1985; Foa and Kozak, 1986; Cuthbert et al., 2003). In PTSD and specific phobia, excessive retrieval of aversive memories causes re-experiencing symptoms and may reinforce negative beliefs, which may increase the avoidance behavior during exposure therapy (Rapee and Heimberg, 1997; Fehm and Margraf, 2002; de Quervain and Margraf, 2008). Interestingly, psychobiological approaches have recently been developed with the aim to reduce excessive retrieval of aversive memories in patients with AD in the context of an exposure-based treatment (de Quervain and Margraf, 2008). It is demonstrated that glucocorticoid administration during exposure enhances the effectiveness of exposure-based treatments in PTSD and specific phobia (Soravia et al., 2006; de Quervain et al., 2011). The exact mechanisms underlying the beneficial effect of glucocorticoids on exposure therapy benefit remain elusive. However, the administration of glucocorticoids during exposure therapy might modulate fear retrieval and thus interrupt the vicious cycle of spontaneous retrieving, re-experiencing, and reconsolidating of aversive memories in phobic patients leading to a reduction of maladaptive fear responses (de Quervain and Margraf, 2008; de Quervain et al., 2011). Similarly, the narrative exposure therapy (NET) represents an example of a behavioral intervention, which is aimed to selectively change and reorganize the retrieval of past emotional experiences. NET is a short-term therapy, which is shown to be an effective treatment for PTSD, especially in individuals traumatized by conflict and/or organized violence (Robjant and Fazel, 2010). During NET, PTSD patients are repeatedly exposed to emotional memories of traumatic events and trained to reorganize such high-emotional autobiographical memories into less-emotional coherent chronological narrative. In a typical NET session, patients are asked to narrate stressful life events in a chronological order. Interestingly, it has been shown that patients who are able to form a highly specific and consistent narrative of individual traumatic events benefit most from exposure therapy for PTSD (Foa and Kozak, 1986; Foa et al., 1995; Robjant and Fazel, 2010).

In sum, evidence so far suggests that valuable information on the characteristics of episodic memory alterations in various AD should be taken into consideration when designing and implementing a psychological treatment plan.

## Concluding Remarks

The importance of episodic memories in the context of AD and other mental disorders is increasingly recognized in the last decades. We have discussed several methodological factors that warrant careful consideration in future research on episodic memory functions in clinical populations. We further provided arguments why clinical researchers should incorporate valid paradigms for the assessment of episodic memory functions in order to better understand the pathology and to optimize psychological treatment. The episodic memory system allows the storing and recollecting of emotional experiences and anticipation of potential emotional events in the future. The complexity of everyday episodic memory can thus not be captured through the use of verbal learning tests or other similar standardized memory tests currently used in the clinical setting. Both the recollection of past emotional experiences and the anticipation of future events can affect patient’s behavior as well as patient’s response to current therapeutic interventions. A major task for psychotherapists and other clinicians is to develop novel research methods and training programs that might help to understand, interpret, and possibly actively use patient’s episodic memory to support therapeutic interventions and alleviate the severity and frequency of symptoms. Finally, more research is needed to understand the underlying neuronal basis of episodic memory alterations in AD.

## Conflict of Interest Statement

The authors declare that the research was conducted in the absence of any commercial or financial relationships that could be construed as a potential conflict of interest.
